# Impact of cardiac arrest duration on extravascular lung water and pulmonary vascular permeability in patients with postcardiac arrest syndrome: a prospective observational study

**DOI:** 10.1186/cc9560

**Published:** 2011-03-11

**Authors:** T Tagami, R Tosa, M Omura, J Hagiwara, N Kido, H Hirama, H Yokota

**Affiliations:** 1 Aidu Chuo Hospital, Aizuwakamatsu, Fukushima, Japan; 2 Nippon Medical University, Bunkyo, Japan

## Introduction

Pulmonary dysfunction after cardiac arrest is a common phenomenon. Evidence appears to support the usefulness of quanti-tative assessment of pulmonary dysfunction using extravascular lung water (EVLW) and the pulmonary vascular permeability index (PVPI). We hypothesized that the duration of cardiac arrest (CPA TIME) would impact the pulmonary dysfunction in patients with postcardiac arrest syndrome. The aim of the present study was to investigate the lung dysfunction quantitatively using EVLW and PVPI in successfully resuscitated patients after cardiac arrest (CPA).

## Methods

This was a prospective observational study of 106 (59 male, 47 female) postcardiac arrest syndrome patients. Eligible patients included all who were in CPA on arrival at the hospital and experienced effective resuscitation resulting in resumption of spontaneous circulation. All patients were resuscitated per therapeutic protocol in our hospital. The CPA TIME from the scene was recorded. The patients were divided into two groups by the cause of CPA; cardiogenic (CG) or noncardiogenic (NCG). Thermodilutional EVLW and PVPI measurements were performed using the PiCCO monitoring system (Pulsion Medical Systems, Munich, Germany) as soon as the patients were admitted to the ICU.

## Results

A moderate positive correlation was documented between the CPA TIME, EVLW (*r *= 0.36, *P *< 0.001) and PVPI (*r *= 0.43, *P *< 0.001) in all 106 patients. In the CG group, we found a very close positive correlation between the CPA TIME, EVLW (*r *= 0.52, *P *< 0.001) and PVPI (*r *= 0.75; *P *< 0.001). No correlations were documented between the CPA TIME, EVLW (*r *= 0.25, *P *= 0.05) and PVPI (*r *= 0.24, *P *= 0.06) in the NCG group.

## Conclusions

The duration of cardiac arrest impacts on the increase in the EVLW and PVPI, especially in patients with CG postcardiac arrest syndrome.

